# Case Report: Heparin-induced thrombocytopenia following double filtration plasmapheresis in a patient with anti-GAD65 autoimmune encephalitis

**DOI:** 10.3389/fcvm.2025.1574698

**Published:** 2025-04-10

**Authors:** Ying Chen, Wanwan Li, Liping Ni, Yufang Mei, Yan Zhou, Wenbin Wan

**Affiliations:** Department of Neurology, Renji Hospital, Shanghai Jiao Tong University School of Medicine, Shanghai, China

**Keywords:** heparin-induced thrombocytopenia, autoimmune encephalitis, double filtration plasmapheresis, heparin, platelet, thrombus

## Abstract

Autoimmune encephalitis (AE) is a group of disorders characterized by antibodies targeting neuronal cell surface, intracellular structures and synapse antigens. Treatment for AE involves reducing antibody levels and suppressing immune-mediated inflammation using intravenous immunoglobulin, plasma exchange (PE), and immune-modulating agents. PE is commonly used in autoimmune neurological diseases, but the safety issues of PE are worth continuous attention. This case report describes a 28-year-old patient who was diagnosed with anti-GAD65 AE and underwent treatments including double filtration plasmapheresis (DFPP), steroids, and immunosuppressive agents. However, complications arose when the patient developed thrombosis and was diagnosed with type II heparin-induced thrombocytopenia (HIT). He was treated with an oral anticoagulant and eventually recovered. One month later, follow-up examinations showed no presence of emboli and his epilepsy remained well controlled. There is a risk of HIT, a potentially dangerous adverse reaction to heparin during treatment of PE. The current case highlights the importance of monitoring for HIT during PE and the need for alternative anticoagulants.

## Introduction

Autoimmune encephalitis (AE) is a group of disorders characterized by antibodies targeting antigens on the neuronal cell surface and synapse. The clinical presentation of AE can vary widely, with symptoms including memory impairment, epileptic seizures, abnormal mental and behavioral functions, and specific subtype-related features (1, 2). Symptoms may develop suddenly or progress slowly over time, posing diagnostic challenges due to the diverse changes in clinical presentation.

Various types of AE antibodies have been identified, including anti-N-Methyl-D-Aspartate (NMDA) receptor antibody, anti-γ-Aminobutyric acid sub-type A (GABAA) receptor antibody, anti-GABA sub-type B (GABAB) receptor antibody, and anti-glutamic acid decarboxylase (GAD) antibody (2). These antibodies directly or indirectly cause inflammatory damage to the central nervous system (CNS), leading to AE development. Treatment strategies for AE focus on reducing antibody levels and suppressing immune-mediated inflammatory damage (3). Commonly used treatments include intravenous immunoglobulin (IVIG), plasma exchange (PE), and immune-modulating agents (4). PE is effective in removing disease mediators from the body and has been widely used in autoimmune neurological diseases. Recent evidence suggests that PE is a suitable option for AE, especially when steroids or other immunosuppressive therapies are not effective or contraindicated in the short term (5, 6). According to the guidelines for the use of therapeutic apheresis in clinical practice (7), PE is recommended as the initial treatment for NMDAR encephalitis, with a recommendation of Category I (7).

Administration of an anticoagulant is essential during PE to prevent clotting within the circuit and ensure optimal treatment efficacy (8). Unfractionated heparin (UFH) is commonly used as an anticoagulant due to its clinical safety. However, it is crucial to be aware of heparin-induced thrombocytopenia (HIT) as a rare but potentially life-threatening adverse drug reaction to heparin in patients undergoing PE (9). Our current case report documents a patient who developed HIT type II and extensive thrombosis after undergoing double filtration plasmapheresis (DFPP) to treat anti-GAD65 AE.

## Case report

This case report has been approved by the Ethics Committee of Renji Hospital, School of Medicine, Shanghai Jiao Tong University. In April 2023, a 28-year-old man with a history of limb convulsions and impaired consciousness presented to our institution's Department of Neurology. These symptoms had been occurring for the past 2 years and were accompanied by upward eye rolling. The seizures lasted for about 1 min and could end on their own. Two years ago, when the symptoms first appeared, he was diagnosed with epilepsy at another medical institution and was prescribed sodium valproate and perampanel. During that time, abnormal signals were found in the right frontal lobe and thalamus on a brain MRI, but no abnormalities were found in the cerebrospinal fluid (CSF) examination. Despite the medication, his epilepsy remained poorly controlled. The patient had been in good health previously, without chronic diseases such as diabetes and hypertension, and without any autoimmune diseases.

**Figure 1:**
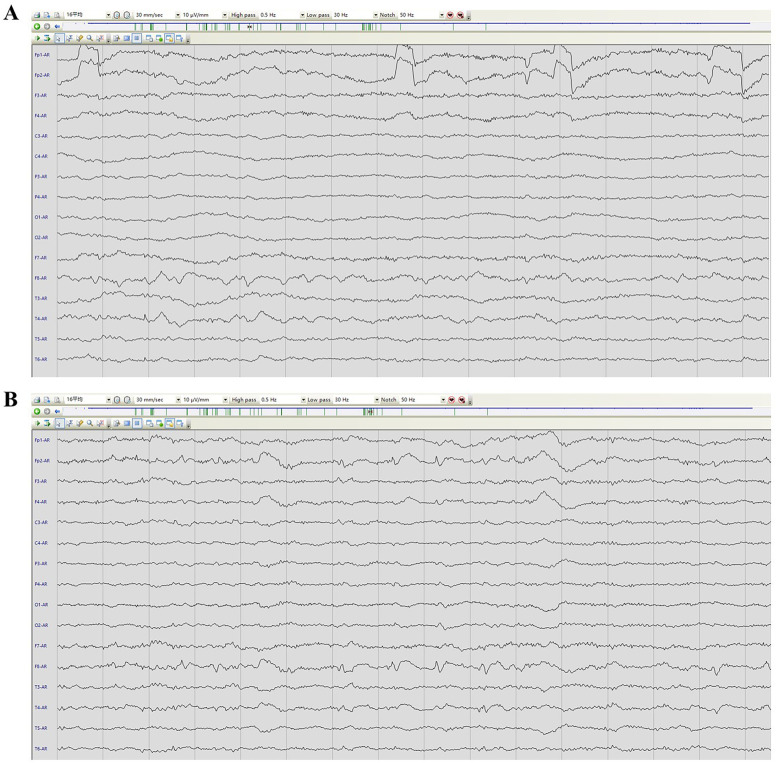
EEG image of the patient. **(A)** presented an awake EEG with sharp wave slow wave. **(B)** showed a sleep EEG with sharp waves and slow waves.

**Figure 2:**
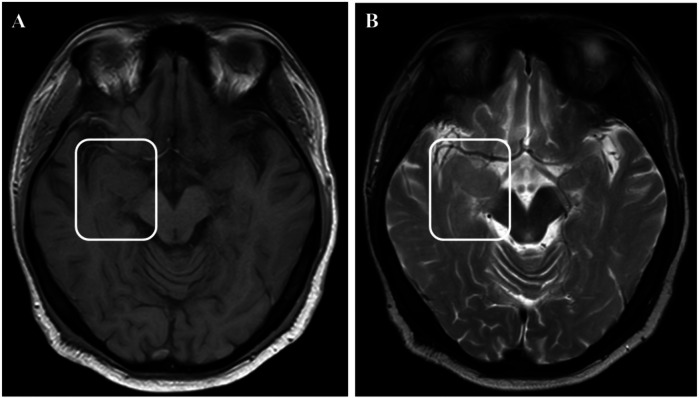
MRI images showed enlargement of the right amygdala and hippocampi. **(A)** showed T1 weight image. **(B)** showed T2 weight image**.**

**Figure 3:**
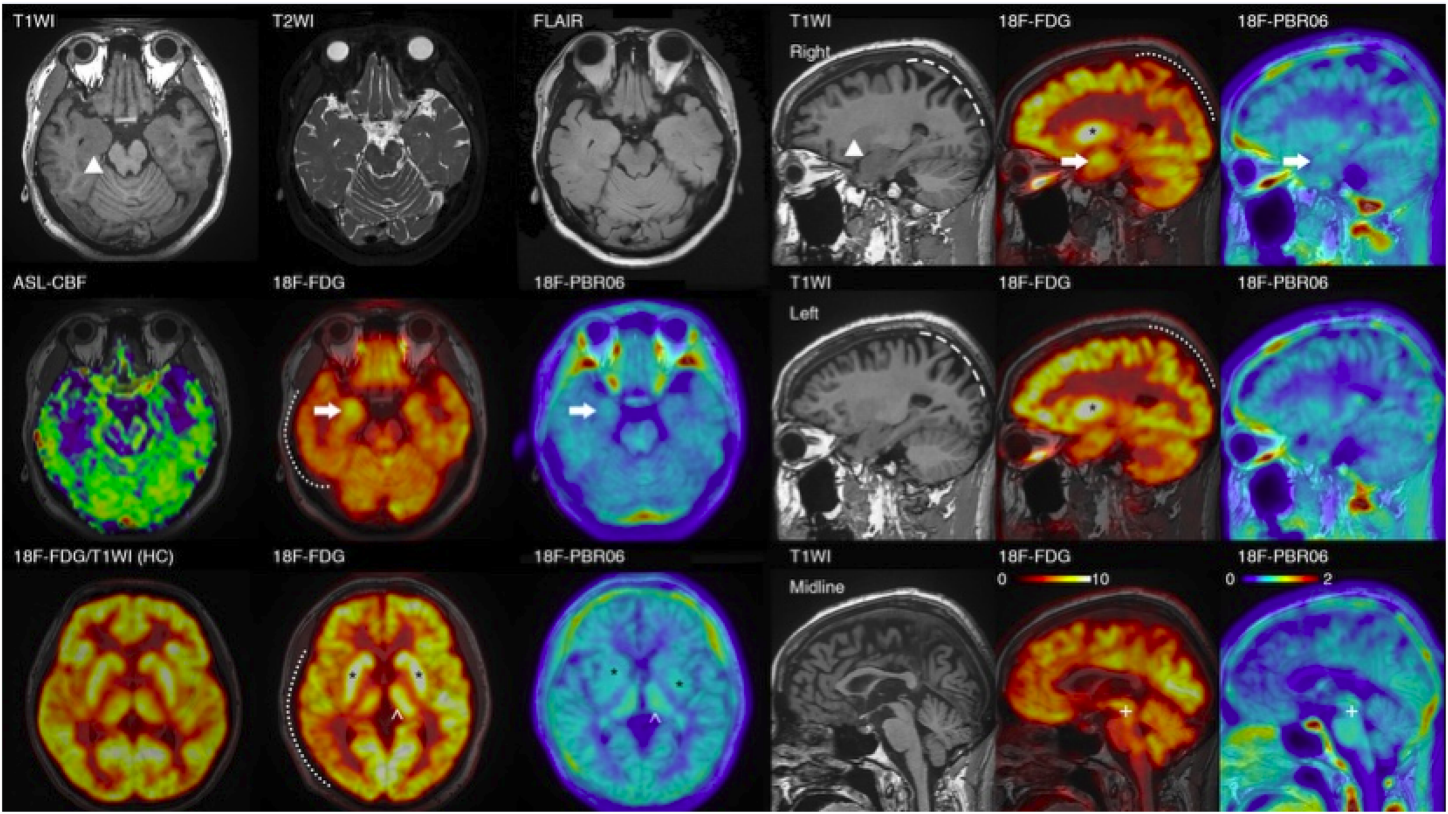
PET/MRI images of the patient. The PET/MRI imaging displays decreased FDG absorption in the cortex of the right hemisphere and the left parietal lobe (indicated by a dotted line). It also shows atrophy in both parietal lobes (marked by a dashed line) in the patient with encephalitis. Regarding focal changes, the volume of the hippocampus on both sides has increased (▴), with heightened FDG and TSPO uptake (→), particularly on the right. However, no noticeable abnormalities were detected in T2\FLAIR\ASL scans. Additionally, there is increased FDG and TSPO uptake in the bilateral basal ganglia (*), and the left thalamus (^). (In comparison with the bottom-left control: the uptake in the cortex and basal ganglia are essentially equivalent, but the patient's basal ganglia-to-cortex ratio is markedly elevated); the FDG\TSPO uptake in the midbrain is also enhanced (+).

**Figure 4:**
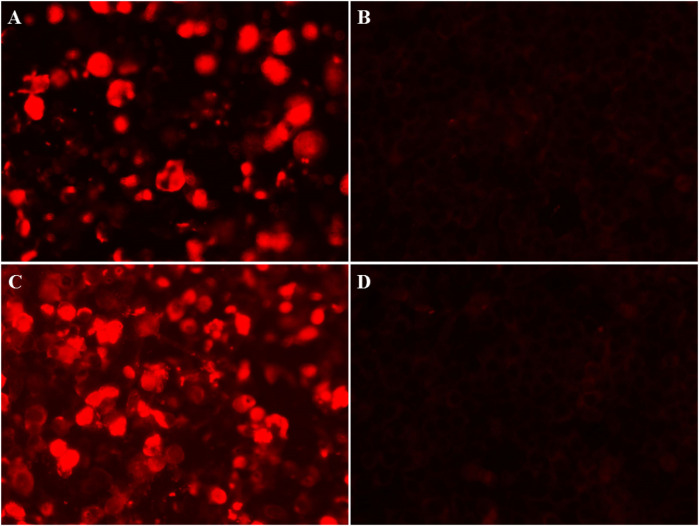
Immunofuorescence images in CSF and serum of anti-GAD65 antibody. **(A)** Positive reaction in CSF of the patient was observed in fixed HEK293 cells, with a titer of 1:30. **(B)** A negative control in CSF. **(C)** A positive reaction was detected in the serum, with a titer of 1:100. **(D)** A negative control in serum.

Physical examination during his hospitalization revealed no abnormal findings in his cognitive function. CSF tests showed normal cell count, protein, glucose, and chloride levels. Long-term electroencephalogram (EEG) examination revealed epileptiform electrical discharges in the right frontal and anterior temporal areas, and slowing waves in the right frontal and the temporal areas (Figure 1). Magnetic resonance imaging (MRI) of the brain showed enlargement of the right amygdala and hippocampi (Figure 2). To further evaluate changes in the brain, positron emission tomography/magnetic resonance imaging (PET/MRI) scans using 18F-FDG and TSPO were performed. These scans showed increased volume, metabolism, and TSPO uptake in the medial temporal lobes. Increased metabolism was also observed in the basal ganglia, left thalamus, left temporoparietal junction, midbrain, and localized cerebellum on the right. In contrast, decreased metabolism was observed in the right cerebral hemisphere and left parietal lobe. Additionally, increased TSPO uptake was detected in the left localized frontal lobe, bilateral insular lobes, and both medial temporal lobes (Figure 3). The PET/MRI scans did not detect any tumors, and no tumor markers were found in the patient's serum. Anti-GAD65 IgG antibodies were detected in both the serum and CSF, with antibody titers of 1:100 and 1:30, respectively (Figure 4).

Based on these findings, the patient was diagnosed with anti-GAD65 AE. He underwent treatments including double filtration plasmapheresis (DFPP) via a femoral vein, intravenous methylprednisolone (IVMP), oral steroids, and the long-term immunosuppressive agent mycophenolate mofetil (MMF). DFPP was performed every other day, filtering 3,300 ml of plasma per session. Membrane filtration type of DFPP was performed every other day, with a total of 3 sessions, filtering 3,300 ml of plasma per session. Plasauto Σ (Asahi Kasei Medical, Tokyo, Japan). Plasmaflo (OP-08W, Asahi Kasei Medical, Tokyo, Japan) and Cascadeflo EC (EC-20W or EC-30W, Asahi Kasei Medical, Tokyo, Japan) were used as the plasma separator and plasma component separator, respectively.

**Figure 5:**
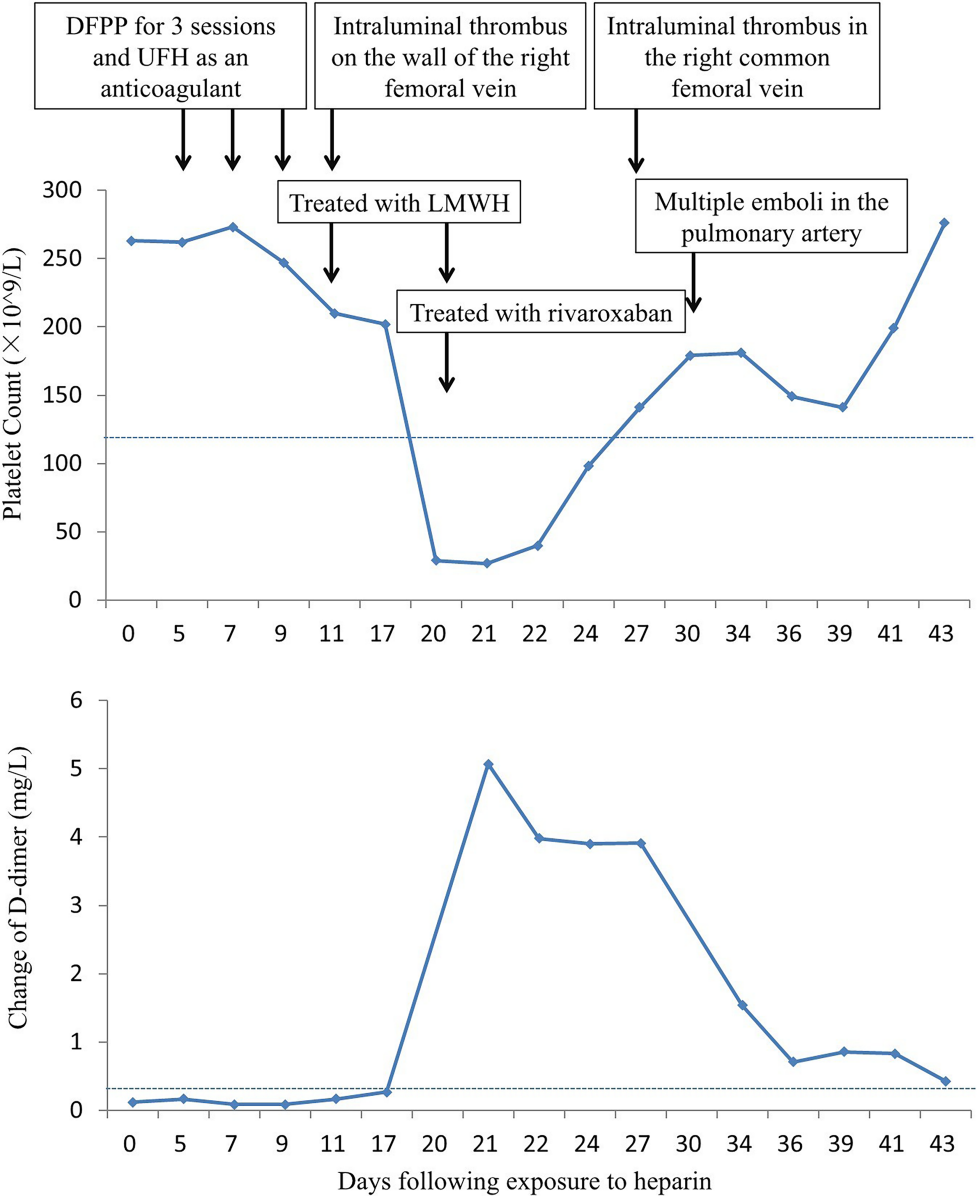
Changes of plate count and D-dimer level.

**Figure 6:**
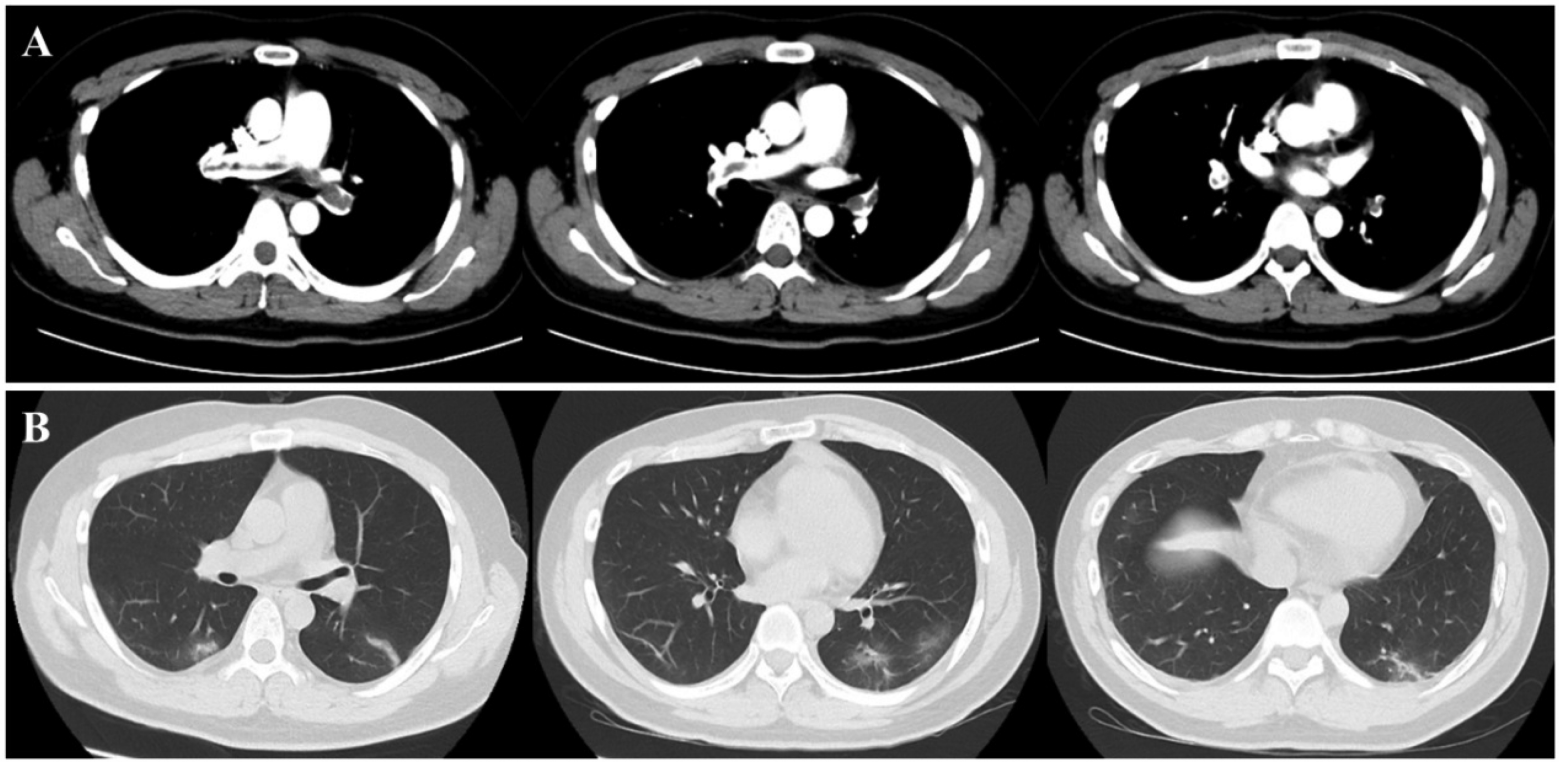
Present of thrombus in pulmonary veins and inflammatory changes in lung. The chest CT scan revealed the presence of multiple emboli in the pulmonary artery **(A)**, as well as inflammation **(B)** in the lower lobes of both lungs.

After the third session of DFPP, the patient's coagulation report indicated a lack of clotting, leading to the discontinuation of his apheresis treatment. An intraluminal thrombus was found on the wall of the right femoral vein, and the patient was subsequently treated with low molecular weight heparin (LMWH). However, one week later, the patient developed a high fever (38.8°C) and his platelet count dropped dramatically from 202 × 10^9^/L to 27 × 10^9^/L (Figure 5). D-dimer levels also increased during the same period Figure 5. Ultrasound examination revealed the formation of a blood clot in the right common femoral vein, and a computed tomography (CT) scan detected multiple emboli in the pulmonary artery, as well as inflammation in the lower lobes of both lungs (Figure 6). The patient's 4 T score indicated a high probability of heparin-induced thrombocytopenia (HIT), and he tested positive for heparin-PF4 antibodies by ELISA.

As a result, the patient was prescribed the oral anticoagulant rivaroxaban to manage the thrombosis. He also received a platelet transfusion and antibiotic agents. After about a week, his platelet count returned to normal, and his fever, pulmonary inflammation, and pulmonary thrombosis gradually improved. One month later, follow-up examinations including CT and ultrasound showed no presence of emboli in his pulmonary artery or femoral vein, respectively. His epilepsy remained well controlled, and he has not experienced any seizures since then.

## Discussion

HIT is a serious complication that can occur when exposed to any form or amount of heparin products. It is also the most frequent non-bleeding complication associated with exposure to UFH. For this patient, except the use during DFPP, LMWH was also used after the intraluminal thrombus on the femoral vein was found, which was almost the same time with clotting deficiency. And pulmonary inflammation and thrombocytopenia occur almost simultaneously. Considering the most characteristic feature of HIT is that it occurs between 5 and 10 days after the start of heparin therapy. We tend to think that infection and independent LMWH as the confounding factors may have facilitated the occurrence of the result, but the use in the DFPP process may play a major role. This condition is characterized by a decrease in platelet counts and an increased propensity for clotting (10). Up to 5% of patients receiving therapeutic doses of heparin may develop HIT, with up to 50% experiencing thromboembolic complications (11). HIT can lead to life-threatening blood clotting, resulting in significant morbidity and mortality rates. It is reported that the mortality rate of patients with HIT during their hospital stay is four times higher compared to those diagnosed with other causes of thrombocytopenia (12). Additionally, patients with HIT have a median length of stay in the hospital that is three times longer, and the cost of their hospitalization is four times higher (12).

The pathophysiology of HIT is complex. Type I HIT is caused by non-immune mechanisms and is more common than Type II HIT, which is an immune reaction mediated by antibodies (13, 14). In Type II HIT, the presence of platelet factor 4 (PF4) binding to heparin triggers the production of antibodies that attack the heparin-PF4 complex, leading to platelet activation via FcγRIIa and severe clot formation (15, 16). However, diagnosing HIT can still be challenging to diagnose and manage and requires the integration of clinical symptoms and laboratory testing.

The current guidelines recommend that when there is suspicion of moderate or high 4Ts score for HIT, exposure of heparin should be immediately discontinued and non-heparin alternative therapy should be used (17). Notably, alternative anticoagulant therapies should be initiated early. It is recommended that direct oral anticoagulants (DOACs), such as direct thrombin inhibitors (DTIs, e.g., argatroban) (18) or Factor Xa inhibitors (e.g., rivaroxaban) (19) could be used in patients who are clinically stable. A recent study has shown that DOACs are safe and effective for the treatment of HIT, suggesting that the use of DOACs in patients with confirmed or suspected HIT is becoming more common (19).

PE is a crucial therapeutic strategy for acute autoimmune disorders such as AE, myasthenia gravis (MG), acute inflammatory demyelinating polyradiculoneuropathy (AIDP), and neuromyelitis optica spectrum disorder (NMOSD) (7). DFPP is a blood purification technique deriving from the TPE modality and is semi-selective in nature (20, 21). It is widely recognized in the medical community that patients who undergo cardiac surgery and receive dialysis treatment for acute kidney injury are at a significantly higher risk of developing HIT compared to the general population in a hospital setting (22). Although it has been reported in the literatures of the development of HIT in patients undergoing therapeutic plasma exchange (TPE) (23–25), no case report has been found in diseases treated with DFPP.

Accumulating evidence suggests that PE is effective and safe in clinical practice. However, striking a balance between the risks of bleeding and thrombosis is essential. Heparin is commonly used as an anticoagulant during PE procedures (26), but Type II HIT is rarely reported in this context. If HIT occurs, discontinuing heparin is crucial (27). However, if the patient's condition allows, PE can still be continued using a non-heparin anticoagulant strategy. PE is also regarded as a highly effective treatment for HIT because it successfully eliminates the immune response against the heparin-PF4 complex and stops the release of prothrombotic factors resulting from platelet aggregation. As a result, it immediately halts the formation of blood clots (18, 28). In addition to coagulation tests during PE, monitoring platelet counts is essential for timely detection of potential abnormalities.

## Conclusion

PE is widely used in clinical practice, especially in neurological autoimmune diseases. Our current patient presented as a typical case of Type II HIT with new-onset thrombocytopenia and extensive venous thrombosis two weeks after heparin exposure during DFPP treatment. Clinicians should be aware of the risk of HIT and the importance of monitoring platelet counts and coagulation parameters during apheresis treatment and exercise caution.

## Data Availability

The datasets presented in this article are not readily available because of ethical and privacy restrictions. Requests to access the datasets should be directed to the corresponding author.
